# Metabolite and lipoprotein responses and prediction of weight gain during breast cancer treatment

**DOI:** 10.1038/s41416-018-0211-x

**Published:** 2018-11-07

**Authors:** Torfinn S. Madssen, Inger Thune, Vidar G. Flote, Steinar Lundgren, Gro F. Bertheussen, Hanne Frydenberg, Erik Wist, Ellen Schlichting, Hartmut Schäfer, Hans E. Fjøsne, Riyas Vettukattil, Jon Lømo, Tone F. Bathen, Guro F. Giskeødegård

**Affiliations:** 1 Department of Circulation and Medical Imaging, Faculty of Medicine and Health Sciences, NTNU–Norwegian University of Science and Technology, P.O. Box 8905 MTFS, Trondheim, 7491 Norway; 2 Department of Oncology, Oslo University Hospital, Oslo, 0424 Norway; 3 Department of Clinical Medicine, Faculty of Health Sciences, UiT The Arctic University of Norway, Tromsø, 9037 Norway; 4 Department of Oncology, St. Olavs University Hospital, P.O. Box 3250 Sluppen, Trondheim, 7006 Norway; 5 Department of Clinical and Molecular Medicine, Faculty of Medicine and Health Sciences, NTNU–Norwegian University of Science and Technology, P.O. Box 8905 MTFS, Trondheim, 7491 Norway; 6 Department of Neuromedicine and Movement Science, Faculty of Medicine and Health Sciences, NTNU–Norwegian University of Science and Technology, Trondheim, 7491 Norway; 7 Department of Physical Medicine and Rehabilitation, St. Olav University Hospital of Trondheim, P.O. Box 3250 Sluppen, Trondheim, 7006 Norway; 8 Department of Breast and Endocrine Surgery, Oslo University Hospital, P.O. Box 4953 Nydalen, Oslo, 0424 Norway; 9 Bruker BioSpin GmbH, Application Method Development Group, Silberstreifen, 76287 Rheinstetten, Germany; 10 Department of Surgery, St. Olavs University Hospital, P.O. Box 3250 Sluppen, Trondheim, 7006 Norway; 11 Institute of Clinical Medicine, Faculty of Medicine, University of Oslo, P.O Box 1171 Blindern, Oslo, 0318 Norway; 12 Division of Paediatric and Adolescent Medicine, Oslo University Hospital, P.O. Box 4956 Nydalen, 0424 Oslo, Norway; 13 Department of Pathology, Oslo University Hospital, P.O. Box 4953 Nydalen, Oslo, 0424 Norway

**Keywords:** Cancer metabolism, Breast cancer, Metabolomics

## Abstract

**Background:**

Breast cancer treatment has metabolic side effects, potentially affecting risk of cardiovascular disease (CVD) and recurrence. We aimed to compare alterations in serum metabolites and lipoproteins during treatment between recipients and non-recipients of chemotherapy, and describe metabolite profiles associated with treatment-related weight gain.

**Methods:**

This pilot study includes 60 stage I/II breast cancer patients who underwent surgery and were treated according to national guidelines. Serum sampled pre-surgery and after 6 and 12 months was analysed by MR spectroscopy and mass spectrometry. In all, 170 metabolites and 105 lipoprotein subfractions were quantified.

**Results:**

The metabolite and lipoprotein profiles of chemotherapy recipients and non-recipients changed significantly 6 months after surgery (*p* < 0.001). Kynurenine, the lipid signal at 1.55–1.60 ppm, ADMA, 2 phosphatidylcholines (PC aa C38:3, PC ae C42:1), alpha-aminoadipic acid, hexoses and sphingolipids were increased in chemotherapy recipients after 6 months. VLDL and small dense LDL increased after 6 months, while HDL decreased, with triglyceride enrichment in HDL and LDL. At baseline, weight gainers had less acylcarnitines, phosphatidylcholines, lyso-phosphatidylcholines and sphingolipids, and showed an inflammatory lipid profile.

**Conclusion:**

Chemotherapy recipients exhibit metabolic changes associated with inflammation, altered immune response and increased risk of CVD. Altered lipid metabolism may predispose for treatment-related weight gain.

## Introduction

Treatment for breast cancer includes surgery, and often also systemic therapies and radiation, all of which are associated with different side effects.^1^ For instance, chemotherapy may have acute toxic effects such as bone marrow suppression and mucositis, as well as various long-term effects. These may vary across different chemotherapeutics, for example, cardiotoxicity for anthracyclines, and peripheral neuropathy and musculoskeletal pain for taxanes.^1–3^ Breast cancer treatment has also long been associated with weight gain during treatment, and may also cause unfavourable changes in body composition, lipid profile and increase the risk of metabolic syndrome.^4–6^ Breast cancer survivors have increased risk of death from cardiovascular disease (CVD), with both chemotherapy and left-sided radiotherapy being associated with increased cardiovascular mortality.^7,8^ Post-diagnosis weight gain and body fat increase have been associated with a worse prognosis and higher risk of regional recurrence.^9,10^ Moreover, an unfavourable metabolic profile is associated with increased mortality and risk of recurrence in breast cancer patients.^11,12^ The adverse metabolic side effects induced by breast cancer therapy may therefore affect breast cancer progression and overall survival.

The mechanisms behind the metabolic side effects of breast cancer treatment are not clear. Suggested mechanisms for post-diagnosis weight gain include physical inactivity during cancer treatment, hormonal changes and changes in metabolic rate.^13^ More recent studies suggest inflammation and oxidative stress as potential mediators between chemotherapy and metabolic syndrome.^4^ A negative metabolic profile may impact disease progression and prognosis through several mechanisms. Increased adipose tissue may lead to increased production of oestrogen, adipokines and other growth factors, leading to a pro-oncogenic environment.^14^

Metabolomics is concerned with high-throughput identification and quantification of metabolites. Metabolic characterization of liquid biopsies, which reflect the systemic effects of the disease and treatment, are gaining increased interest.^15–17^ The metabolome comprises all low-molecular-weight compounds in the human body, and reflects the dynamic interaction between the individual and the environment. Metabolomics can therefore provide insights into metabolic and physiological changes of pathobiological significance during cancer treatment. The aim of this pilot study was to characterize alterations in the metabolite and lipoprotein profiles in serum samples of breast cancer patients undergoing treatment, and to characterize metabolic patterns predisposing for weight gain.

## Materials and methods

### Study population and study design

This pilot study includes 60 breast cancer patients participating in a clinical study which includes physical exercise during breast cancer treatment. Patients were recruited between 2011and 2014 at the Cancer Centre, Oslo University Hospital (OUS), St. Olav University Hospital, Trondheim, and Vestre Viken HF, Drammen. The patients were included at the time of diagnosis with invasive breast cancer or/and ductal carcinoma in situ grade 3, verified by histology. To be included in the study, the patients had to be between 35 and 75 years old. The intervention included 60 min of supervised high-intensity interval training and strength training twice a week, as well unsupervised exercise for 60 min twice a week. Patients were randomized to the intervention or control group by menopausal status, and patients in the two groups were equally distributed in recipients and non-recipients of chemotherapy (49 and 56%, respectively). Women with severe illness, including heart disease, uncontrolled diabetes, prior cancers, breast cancer with stage 3/distant metastases or prior bariatric surgery, were excluded. All patients underwent surgical removal of the tumour, and subsequent treatment regime followed national guidelines from the Norwegian Breast Cancer Group.^18^ The chemotherapy regimens used were either fluorouracil, epirubicine and cyclophosphamide (FEC) every third week for 6 cycles, or 4 cycles of either FEC followed by 12 weeks of either docetaxel every third week or paclitaxel every week. The study was approved by The Regional Committee for Medical and Health Research Ethics (REK 2011/500), and all patients gave informed written consent to participate.

### Assessment of clinical variables

Clinical variables were assessed and venous fasting blood samples were taken three times: at baseline (the time of inclusion: 0–7 days before surgery), and 6 and 12 months after surgery. Weight and height were measured with participants wearing light clothing and no footwear. Weight was measured on an electronic scale, and rounded off to nearest 0.1 kg. Weight gain was defined as a weight increase of 1.5 kg or more after 6 months, as done previously by Keun et al.^19^ Venous fasting samples were collected into serum tubes with no additives, and centrifuged at 3000 rotations per min for 10 min approximately 1 h after collection. The serum was stored at −80 °C until the time of analysis. Haemoglobin A1c (HbA1c), triglycerides and high-density lipoprotein (HDL) were measured in fresh sera at the Department of Clinical Chemistry, OUS, Ullevål (Roche Diagnostics/Cobas Integra 800- Cobas 8000, Mannheim, Germany, www.roche.com). Low-density lipoprotein (LDL) was calculated using Friedewalds formula.

### Magnetic resonance experiments

Magnetic resonance spectroscopy (MRS) analysis was performed for serum samples acquired at baseline and 6 and 12 months after surgery. The samples were slowly thawed at 4° C prior to the nuclear magnetic resonance (NMR) analysis. Then, 150 µl of serum was drawn from each sample, mixed with 150 µl of buffer (D_2_O with 0.075 mM Na_2_HPO_4_, 5 mM NaN, 3.5 mM TSP, pH 7.4), and analysed in 3 mm NMR tubes. NMR analysis was performed on a Bruker Avance III Ultrashielded Plus 600 MHz spectrometer (Bruker BioSpin GmbH, Rheinstetten, Germany), equipped with a 5 mm QCI Cryoprobe. Data acquisition and sample handling was fully automated using a SampleJet with Icon-NMR on TopSpin 3.1 (Bruker BioSpin). Carr–Purcelli–Meiboom–Gill (CPMG) and nuclear Overhauser effect spectroscopy (NOESY) spectra with water pre-saturation were acquired at a temperature of 37 °C. The spectra were Fourier transformed to 128 K after 0.3 Hz exponential line broadening.

Further preprocessing and quantification was done in MATLAB R2013b (The Mathworks, Inc., Natick, MA, USA). Chemical shifts of CPMG spectra were referenced to the left peak of the alanine doublet at 1.47 ppm. The baseline was adjusted by setting the lowest point of each spectrum to zero. The spectral region between 0.29 and 8.53 ppm was normalized to equal total area after removal of the water residual peak at 4.33–5.13 ppm. Metabolites were assigned using Chenomx NMR suite 7.7 (Chenomx Inc., AB, Edmonton, Canada) and the Human Metabolome Database (HMDB). Metabolite peaks from normalized spectra were semi-quantified by integration. For metabolites with more than one resonance, either the mean or the resonance with minimum overlap was used. A total of 30 metabolites were semi-quantified by peak integration (Supplementary Table 1), including two lipid signals from the lipid methyl (–CH_3_) protons (peak at 0.8–0.9 ppm) at the end of fatty acid chains, and lipid methylene (–CH_2_–) protons (peak at 1.55–1.60 ppm) arising from the protons from the β-carbon, respectively, in fatty acids mainly from triglycerides and esterified cholesterol within the lipoprotein particles. These lipid signals are referred to as lipid1 and lipid2, respectively. A representative spectrum with annotated metabolite peaks is shown in Supplementary Figure 1.

Lipoprotein subclassification was performed by Bruker BioSpin (Bruker IVDr Lipoprotein Subclass Analysis^TM^), based on one-dimensional NOESY MR spectra using a partial least-squares regression model.^20,21^ Concentrations of cholesterol (CH), free cholesterol (FC), phospholipids (PL), and apolipoprotein A1 (A1), A2 and B (AB) in plasma, as well as in each of the lipoprotein classes (very-low-density lipoprotein (VLDL), intermediate-density lipoprotein (IDL), LDL and HDL), were estimated using a regression model developed by Bruker BioSpin. Additionally, each lipoprotein class was further subdivided into subfractions according to their density. VLDL was divided into VLDL 1–6, LDL into LDL 1–6 and HDL into HDL 1–4, with increasing density, and their concentrations of triglycerides (TG), CH, FC, PL, A1, A2 and AB were estimated, yielding a dataset of 105 variables (Supplementary Table 2). Bruker has published prediction errors for the model in comparison to ultracentrifugation.^22^ VLDL 6 was excluded from the analysis due to poor model reliability. Four-letter abbreviations were used. For example, estimated VLDL4 contents of phospholipids was named V4PL, and estimated total plasma triglycerides was named TPTG.

### Mass spectrometry experiments

Targeted mass spectrometry (MS) analyses were performed on samples from 53 patients taken at baseline and after 6 months, using an Acquity UPLC-1 Class system coupled to a Xevo TQS mass spectrometer (Waters, Milford, MA, USA). The AbsoluteIDQ p180 kit (Biocrates Life Sciences AG, Innsbruck, Austria) was used to quantify 188 different metabolites, which included acylcarnitines, amino acids, phospholipids, sphingolipids and biogenic amines. Amino acids and biogenic amines were measured through liquid chromatography-mass spectrometry, while the remaining metabolites were measured semi-quantitatively by flow-injection analysis mass spectrometry. Amino acids and biogenic amines are given a three-letter abbreviation. Lipid side chains were labelled CX:Y, where X is the number of carbon atoms in the chain and Y is the number of double bonds. Metabolites which had more than 30% missing values or values below the limit of detection (LOD) were excluded from further analysis. This left a dataset of 140 metabolites (Supplementary Table 2). Metabolite concentrations below the LOD were set to LOD/2. To remove outliers caused by instrumental errors, all metabolite concentrations more than 100 times greater than the median value for the metabolite in question were defined as missing values. The coefficients of variation (CoVs) for the quality control measurements were below 15% for all but 5 metabolites, which had CoVs of 15–25%. The CoVs, the number of measurements <LOD and number of measurements >100 times the median value in the data are shown in Supplementary Table 3.

## Statistical methods

### Multivariate modelling

Multilevel OPLS-DA (orthogonal projections to latent structures discriminant analysis) was applied to determine how metabolite levels changed over time in recipients and non-recipients of chemotherapy, respectively.^23,24^ This method is useful for analysing longitudinal data with two multivariate measurements per subject, and can be considered a multivariate analogue of the paired *t*-test. If A is the matrix with measurements from baseline, and B is the matrix with measurements from 6 months/12 months, then time point 1 is represented as A–B, and time point 2 is represented as B–A, in this way focussing the analysis on intrapatient variations. We then perform OPLS-DA to discriminate (A–B) from (B–A). Data from both 6 and 12 months were compared with baseline measurements to characterize systematic time-related changes. OPLS-DA was performed on metabolite and lipoprotein profiles from baseline and 6 months in order to predict weight gain during treatment.

OPLS-DA models were validated using 10-fold cross-validation with 20 iterations. For models where the data contained multiple measurements from each patient, 10% of the patients were left out during each iteration. The number of latent variables was chosen selecting the first local minimum in classification error. Average model sensitivity and specificity over 20 iterations were obtained.

The statistical significance of the models was assessed by permutation testing. Here, the class labels are shuffled to resemble random classification. The *p* value corresponds to the proportion of random classifications giving a result equal to or better than the original model. Permutations were repeated 1000 times, and *p* values of ≤0.05 were considered significant. During interpretation, OPLS-DA loadings were coloured according to VIP (variable importance in projection) score, showing the influence of each metabolite in the classification.^25^

### Univariate statistics

We tested for significant differences in age, menopausal status, tumour characteristics, endocrine therapy, radiotherapy and chemotherapy using a two-sample *t*-test for continuous variables and Fisher’s exact test for categorical variables. Metabolites and lipoproteins were analysed by univariate statistics. The metabolites measured by MS and MRS were analysed using parametric tests, while non-parametric tests were used to analyse the lipoprotein subfractions due to non-normality. Linear mixed models analysis (LMM) was used for modelling time-related trends in individual metabolite concentrations between baseline and 6 months. LMM allows for missing values in paired analyses, ensuring utilization of all data. LMM was performed in R (version 3.4.3, R Foundation for Statistical Computing) using the NLME package. Metabolite concentrations were log-transformed prior to LMM analyses. Changes in lipoprotein subfractions were assessed with Wilcoxon signed-rank tests for recipients and non-recipients of chemotherapy, respectively. Multiple testing correction was performed by false discovery rate (FDR) estimation for the data from MRS, MS and lipoprotein subfractions separately.

MetaboAnalyst 3.0 was used for metabolic pathway analyses of the combined metabolomics data from MS and MRS. Pathway analysis integrates metabolite set enrichment analysis and pathway topology analysis, and may be used to detect subtle, consistent changes in metabolic pathways. Samples from 6 months were compared with baseline samples in chemotherapy recipients and non-recipients separately. In cases with duplicate metabolite measurements, the MS measurements were used. The data were autoscaled prior to analysis. The pathway analysis was performed through a pathway enrichment analysis using a global test and a pathway topology analysis using a relative betweenness centrality measure. Only metabolites with IDs in the KEGG (Kyoto Encyclopedia of Genes and Genomes) could be included, and several acylcarnitines, phosphatidylcholines and sphingolipids were therefore not included in the pathway analysis.

## Results

### Clinical characteristics

An overview of the patient cohort, clinical characteristics and treatment is given in Table 1. There were significant differences in clinical characteristics between recipients and non-recipients of chemotherapy (Table 1). Chemotherapy recipients had lower mean age, larger tumours, more nodal involvement and higher Ki67 levels, and were more likely to receive endocrine therapy. There were no significant differences in serum triglycerides (*p* = 0.62), LDL (*p* = 0.94) or HDL (*p* = 0.12) at baseline. Weight gainers had lower HDL at baseline (*p* = 0.002), but no significant differences were found in triglycerides (*p* = 0.40) or LDL (*p* = 0.51).

**Table 1:** Cohort description

Variable	All patients (*n* = 60)	Chemotherapy (*n* = 35)	No chemotherapy (*n* = 25)	*P* value	Weight gainers (*n* = 17)	Non-weight gainers (*n* = 35)	*P* value
Age							
Mean (range)	55.4 (38–69)	52.8 (38–69)	58.9 (46–68)	0.002	54.1 (42–68)	54.6 (38–69)	0.91
Menopausal status							
Pre	18	14	4	0.053	6	12	*P* > 0.99
Post	42	21	21		11	23	
Hormone receptor status							
Positive	52	31	21	0.29	16	28	*P* > 0.99
Negative	4	4	0		1	3	
DCIS	4	0	4		0	4	
HER2 status							
Positive	3	3	0	0.25	0	3	0.54
Negative	53	32	21		17	28	
DCIS	4	0	4		0	4	
Radiation							
Yes	47	25	22	0.35	14	27	*P* > 0.99
No	13	10	3		3	8	
Surgical procedure (breast conserving surgery/mastectomy/primary reconstruction with expansion)							
BCS	43	22	21	0.08	13	25	0.07
Masectomy	15	12	3		2	10	
PRE	2	1	1		2	0	
Endocrine treatment							
No	20	4	16	<0.001	4	15	0.02
Tamoxifen	13	13	0		5	8	
AI	23	17	6		7	11	
AI+Goserelin	2	1	1		1	1	
Unknown	2	0	2		0	0	
Chemotherapy							
FEC	11	-	-	-	2	9	0.19
FEC+Taxane	24				10	11	
No	25				5	15	
Herceptin							
Yes	3	3	0	0.26	0	3	0.54
No	57	32	25		17	32	
Baseline weight (kg)							
Mean (range)	70.8 (48.5–97.1)	69.4 (48.5–96.6)	72.9 (58.6–97.1)	0.25	70.0 (53.8–97.1)	71.5 (48.5–96.6)	0.65
Baseline HbA1c							
Mean (range)	5.7 (4.5–6.5)	5.7 (5.1–6.3)	5.6 (4.5–6.5)	0.41	5.7 (5.2–6.5)	5.6 (4.5–6.3)	0.26
Weight change (kg)							
Mean (range)	0.67 (−9.80–9.20)	0.70 (−9.80–9.20)	0.62 (−2.80–4.9)	0.92	3.65 (1.50–9.20)	−0.82 (−9.80–1.40)	<0.001

*DCIS* ductal carcinoma in situ, *ER* oestrogen receptor, *BCS* breast conserving surgery, *PRE* primary reconstruction with expansion, *AI* aromatase inhibitor, *FEC* fluorouraicil, epirubicine, cyclophosphamide

### Metabolic changes in patients receiving chemotherapy

**Fig. 1:**
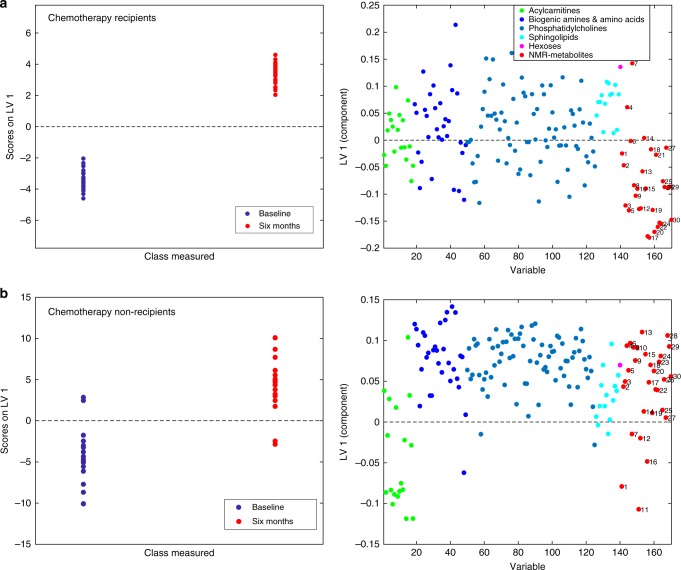
Score and loading plots for multilevel OPLS-DA of **a** chemotherapy recipients and **b** non-recipients. NMR metabolites are labelled: lipid1 (1), leucine (2), valine (3), isoleucine (4), 2-methyl glutarate (5), alanine (6), lipid2 (7), lysine (8), acetate (9), glutamine and glutamate (10), acetoacetate (11), 3-hydroxybutyrate (12), glutamate (13), pyruvate (14), glutamine (15), citrate (16), methionine (17), creatine (18), creatinine (19), ornithine (20), proline betaine (21), dimethyl sulphone (22), unknown (23), histidine (24), glucose (25), glycine (26), lactate (27), tyrosine (28), phenylalanine (29) and formate (30). The figure shows that samples taken after 6 months, having high scores on LV1, have more of the metabolites that are positive on the *y*-loading axes and less of the negative metabolites, compared to samples taken at baseline

**Fig. 2:**
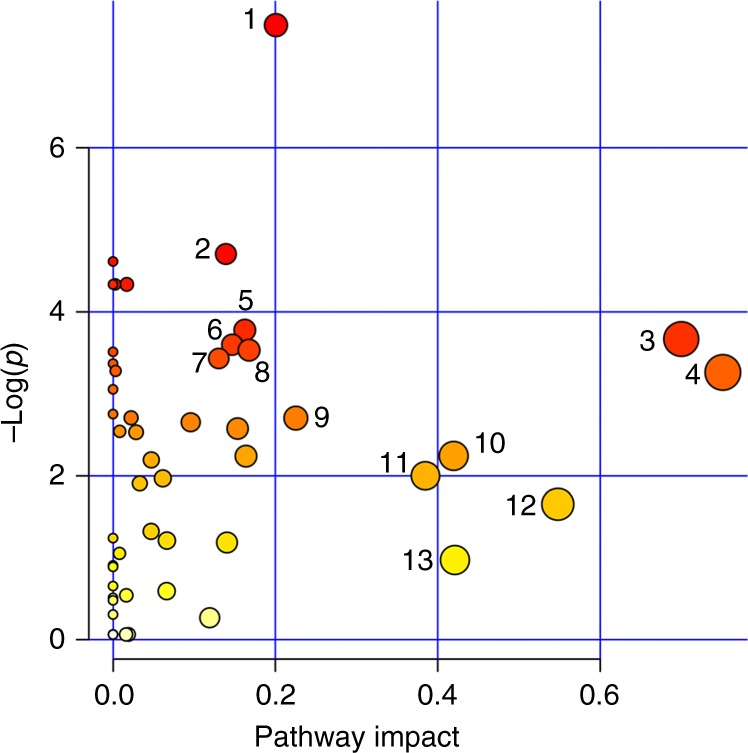
Results from MetaboAnalyst showing altered metabolic pathways in chemotherapy recipients after 6 months. Pathways are labelled: tryptophan metabolism (1), D-glutamine and D-glutamate metabolism (2), synthesis and degradation of ketone bodies (3), alanine, aspartate and glutamate metabolism (4), lysine degradation (5), glyoxylate and dicarboxylate metabolism (6), butanoate metabolism (7), lysine biosynthesis (8), aminoacyl-tRNA biosynthesis (9), pyruvate metabolism (10), taurine and hypotaurine metabolism (11), arginine and proline metabolism (12) and glycine, serine and threonine metabolism (13)

Multilevel OPLS-DA showed that the metabolite profile of chemotherapy recipients was significantly changed after 6 months (accuracy = 91.7%, *p*_perm_ < 0.001). The scores and loadings of the model (Fig. 1a) showed that sphingolipids and lipid2 levels are increased after treatment, while most MRS-measured metabolites other than lipid2 were unchanged or decreased. When testing individual metabolites with LMM (Supplementary Table 2), kynurenine, alpha-aminoadipic acid (alpha-AAA), asymmetric dimethylarginine (ADMA), two phosphatidylcholines (PC aa C38:3, PC ae C42:1) and hexoses were significantly increased after correcting for multiple tests. MRS showed significant increase in lipid2, as well as decreases in 18 different metabolites, 14 of which remained significant after correcting for multiple tests. Multilevel PLS-DA of NMR metabolites showed significant differences also between baseline and 12 months after surgery (accuracy = 75.0%, *p*_perm_ < 0.001). Score and loading plots showed increased levels of acetate, lipid2 and proline betaine, and reduced levels of methionine, tyrosine, ornithine and citrate 12 months after surgery (Supplementary Figure 2). Pathway analysis by MetaboAnalyst showed that after FDR correction, tryptophan metabolism was significantly altered after 6 months (*q* = 0.028, Fig. 2, Supplementary Figure 3A).

**Fig. 3:**
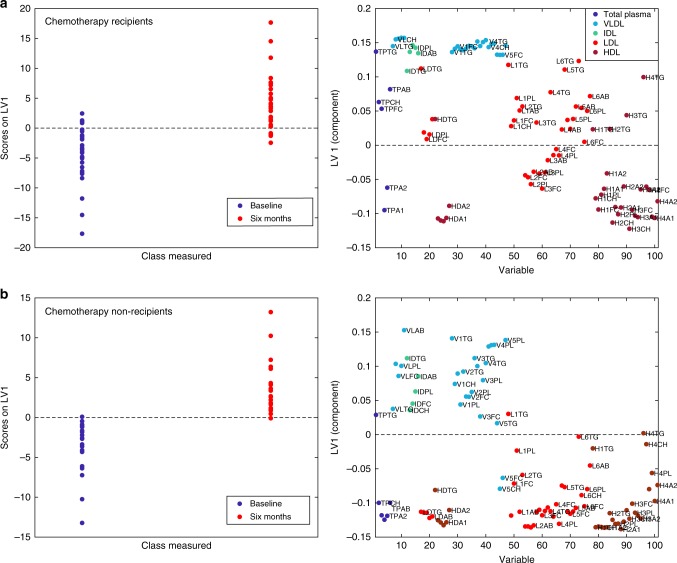
Score and loading plots for multilevel OPLS-DA of **a** chemotherapy recipients and **b** non-recipients. See Materials and methods section for lipoprotein nomenclature. In (**a**), lipids and apolipoproteins in VLDL and LDL 4–6 are increased, while those associated with HDL are decreased, except for triglycerides. In (**b**), no triglyceride enrichment is seen, and LDL-associated lipids and apolipoproteins are decreased

**Fig. 4:**
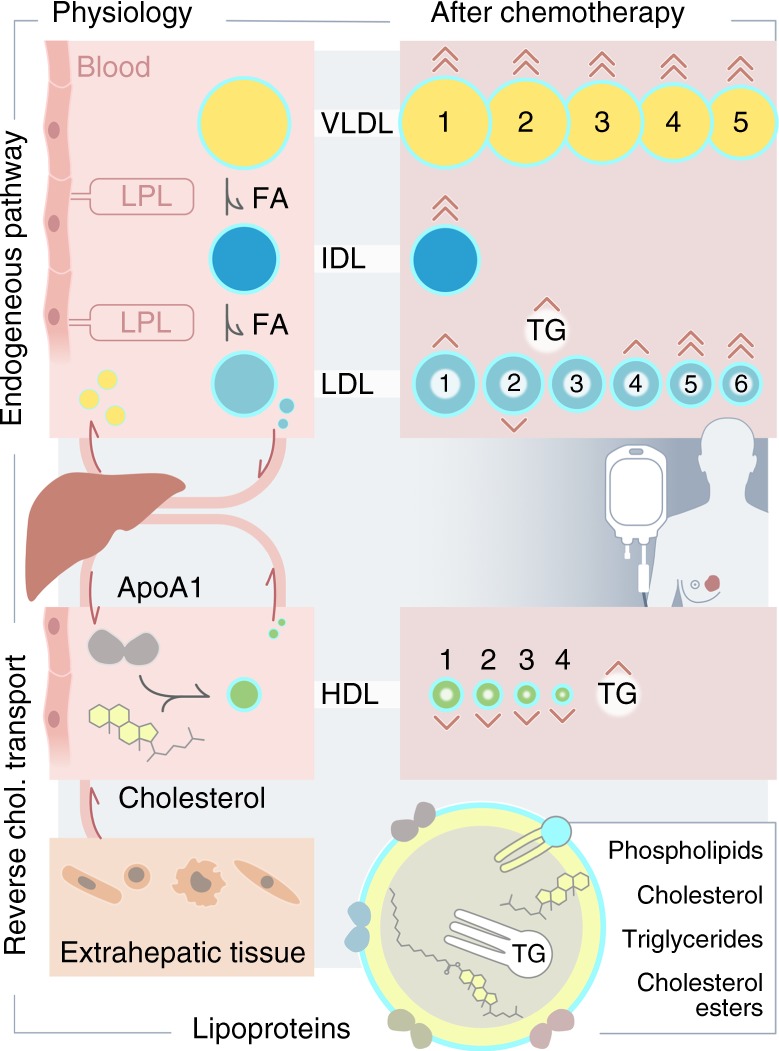
Illustration of lipoprotein alterations in chemotherapy recipients after 6 months. Lipoprotein metabolism subsequent to lipid absorption into the body consists of two interacting pathways. The endogenous pathway supplies lipids to tissues through VLDL, which is hydrolyzed sequentially into IDL and LDL by lipoprotein lipase (LPL), providing fatty acids (FA) to tissues. The reverse cholesterol transport pathway transports cholesterol back to the liver and to other lipoproteins by HDL. In chemotherapy recipients, VLDL, IDL and small LDL particles are increased, while HDL particles are decreased after 6 months. There is also triglyceride (TG) enrichment of LDL and HDL. The directions and number of arrowheads indicate the direction and magnitude of the change for each lipoprotein subclass. Figure by Ellen Tenstad/Science Shaped

Multilevel OPLS-DA showed that chemotherapy recipients experienced significant changes in lipoprotein profiles after 6 months of treatment (accuracy = 80.0%, *p*_perm_ < 0.001). Score and loading plots (Fig. 3a) showed pronounced increases in lipids and apolipoproteins associated with VLDL, IDL and LDL 5–6, especially triglycerides. Lipids and apolipoproteins associated with HDL subfractions were decreased, with the exception of triglycerides, which were increased. Univariate tests showed a highly significant increase in total plasma triglycerides, as well as lipids and apolipoproteins associated with VLDL, IDL and LDL 4–6 after 6 months (Supplementary Table 2). Lipids and apolipoproteins in HDL 2–4 decreased significantly, except for their triglyceride content, which was increased. These results remained significant after correcting for multiple tests. Lipoprotein alterations in chemotherapy recipients are illustrated in Fig. 4. Standard laboratory measurements showed a significant increase in triglycerides (*p* = 0.002), a significant decrease in HDL (*p* = 0.003) and no significant change in LDL (*p* = 0.327). Chemotherapy recipients still had a significantly different lipoprotein profile from baseline 12 months after surgery (accuracy = 78.3%, *p*_perm_ < 0.001). Score and loading plots showed increases in lipids and apolipoproteins associated with LDL 5–6, as well as increases in VLDL- and HDL 3–4-associated lipids and apolipoproteins (Supplementary Figure 4A).

### Metabolic changes in patients not receiving chemotherapy

During the 6 months after surgery, the metabolite profiles of patients not receiving chemotherapy also changed significantly (accuracy = 77.5%, *p*_perm_ < 0.001). The score and loading plots showed that most amino acids, biogenic amines and phosphatidylcholines increased, while levels of acylcarnitines, lipid1 and acetoacetate decreased (Fig. 1b). Individual metabolite levels were tested with LMM, and only glutamate remained significantly increased after 6 months after correcting for multiple tests (Supplementary Table 2). After 12 months, there was no significant difference from the baseline MRS metabolite profile (accuracy = 64.3 %, *p*_perm_ = 0.12). Pathway analysis by MetaboAnalyst did not yield any significantly altered metabolic pathways after FDR correction (Supplementary Figure 3B).

The lipoprotein profiles of patients not receiving chemotherapy had changed significantly 6 months after surgery (accuracy = 82.0%, *p*_perm_ < 0.001) compared to baseline measurements. The score and loading plots showed that most lipids and apolipoproteins in VLDL subfractions increased (Fig. 2b), except for V5FC and V5CH. Lipids and apolipoproteins associated with LDL and HDL subfractions decreased. Univariate tests also showed significant decreases in TPFC, TPA1, TPA2 and IDAB, in addition to significant decreases in lipids and apolipoproteins associated with LDL2 and HDL 2–3, before but not after correction for multiple tests. Standard laboratory measurements showed no significant changes in neither triglycerides and LDL nor HDL. Lipoprotein profiles in patients not receiving chemotherapy remained significantly different from baseline also after 12 months (accuracy = 73.8%, *p*_perm_ < 0.001). Score and loading plots showed increases in most VLDL-associated lipids and apolipoproteins, and decreases in LDL- and HDL-associated lipids and apolipoproteins. Triglycerides in LDL and HDL were less decreased than other lipoprotein contents (Supplementary Figure 4B).

### Metabolite profile of patients with weight gain

**Fig. 5:**
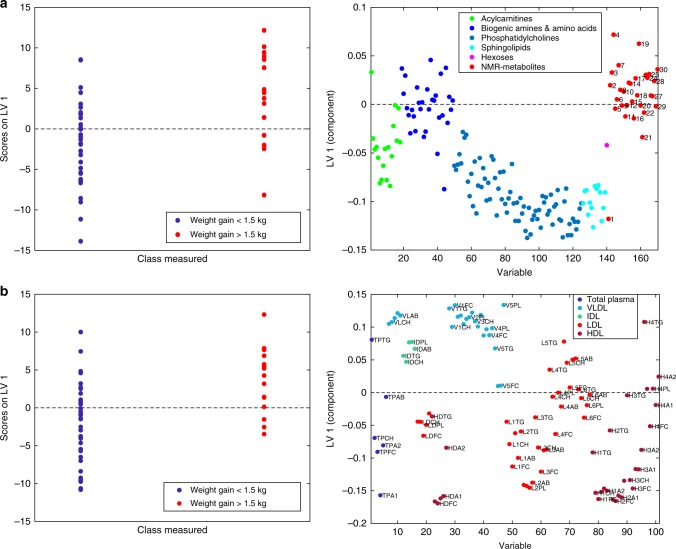
Score and loading plots for OPLS-DA for prediction of weight gain >1.5 kg, from **a** metabolites and **b** lipoproteins, respectively. An inflammatory lipoprotein pattern may also be seen here in (**b**), with lipids and apolipoproteins in VLDL increased, while those in LDL 1–3 and HDL 1–3 are decreased. Triglyceride enrichment of HDL can also be seen

Patients who gained weight had a significantly different metabolite profile at baseline compared to patients who did not gain weight (accuracy = 65.9%, *p*_perm_ = 0.019). Score and loading plots showed that patients who later gained weight had lower levels of acylcarnitines, phosphatidylcholines, sphingolipids and lipid1 (Fig. 5a). At baseline, univariate analyses showed that 3 acylcarnitines, 20 phosphatidylcholines and SM C24:1 had significantly lower levels in patients who later gained weight before but not after correcting for multiple tests (Supplementary Table 2).

We also found significant differences between the baseline lipoprotein profiles of patients who later gained weight and the patients who did not (accuracy = 66.3%, *p*_perm_ = 0.008). Multivariate analysis by PLS-DA showed that patients who later gained weight had higher concentrations of VLDL- and IDL-associated lipids and apolipoproteins at baseline, and lower concentrations of lipids and apolipoproteins in LDL1-3. and HDL1-3 (Fig. 5b). Univariate tests showed significantly lower levels of lipids and apolipoproteins in HDL 1–2 in patients gaining weight, as well as in total HDL, after correcting for multiple tests (Supplementary Table 2).

Patients with weight gain had a significantly different metabolite profile compared to patients not gaining weight also after 6 months (accuracy = 66.1%, *p*_perm_ = 0.014). Score and loading plots showed that patients who had gained weight had higher levels of acylcarnitines, isoleucine and lipid2 (Supplementary Figure 5).

After 6 months, there was no significant difference between the lipoprotein profiles of patients who gained weight, compared to patients who did not gain weight (*p*_perm_ = 0.454).

## Discussion

In this pilot study, we observed that distinct changes in metabolite and lipoprotein profiles occur during breast cancer treatment, both for recipients and non-recipients of chemotherapy. Furthermore, patients who gained weight during treatment had a significantly different baseline metabolite and lipoprotein profile from patients not gaining weight, and a significantly different metabolite profile 6 months after surgery.

Kynurenine levels were increased 6 months after surgery in both patients receiving and not receiving chemotherapy (38 and 25% increases, respectively), but was only significant in chemotherapy recipients. Kynurenines are synthesized from tryptophan by tryptophan 2,3-dioxygenase, and by indolamine-2,3-dioxygenase 1 and 2, which are induced by inflammation. Interestingly, tryptophan metabolism was also found to be significantly altered by MetaboAnalyst. Kynurenines have immunomodulating properties suggested to contribute to the development of tumour immunoresistance.^26^ They may also contribute to the pathogenesis of some mental disorders, including depression, cognitive dysfunction and central fatigue, as well as metabolic and inflammatory disorders.^27,28^ Chemotherapy-induced inflammation has been suggested to be a contributing factor to development of drug resistance, through inducing stromal production of cytokines and growth factors in the tumour.^29^ Kynurenine may therefore represent an additional route through which chemotherapy-induced inflammation may affect morbidity, cognition, and risk of recurrence in breast cancer patients.

Levels of ADMA were increased 6 months after surgery in chemotherapy recipients. ADMA may contribute to vascular dysfunction through competitively inhibiting the oxidation of arginine to citrulline, thereby hindering nitric oxide production. ADMA levels are elevated in various conditions associated with vascular dysfunction, such as diabetes,^30^ heart failure,^31^ preeclampsia,^32^ and in inflammation,^33^ and has therefore been considered a potential biomarker for cardiovascular disease.^34^ ADMA has been shown to attenuate starvation-induced apoptosis in vitro, and is therefore suggested to contribute to blocking apoptosis in response to chemotherapy.^35^ Increased ADMA levels after chemotherapy could therefore lead to increased risk of both CVD and recurrence in breast cancer survivors. However, long-term data on ADMA levels in breast cancer patients are lacking. A previous study on 19 patients with stage 2/3, lymph node-positive breast cancer showed that ADMA levels decreased in breast cancer patients after chemotherapy with taxotere, epirubicin and cyclophosphamide.^36^ Contrary to this, ADMA levels have been shown to be chronically elevated in survivors of acute lymphoblastic leukaemia, suggesting an increased risk of cardiovascular disease.^37^ Thus, ADMA levels after breast cancer treatment need further investigation.

Alpha-AAA and hexose levels were significantly increased in chemotherapy recipients after 6 months. Alpha-AAA is a product of lysine degradation, and higher levels have been suggested as a predictive biomarker for development of diabetes.^38^ Elevated hexose levels could not be explained by increased glucose levels, as neither glucose nor HbA1c were significantly elevated after 6 months in chemotherapy recipients.

Chemotherapy recipients underwent significant changes in lipid metabolism during treatment. VLDL- and LDL 5–6-associated lipids and apolipoproteins were increased, HDL and LDL particles showed triglyceride enrichment and levels of sphingolipids were increased. Additionally, chemotherapy recipients had increased lipid2 signals. Lipid2 originates from lipid methylene (–CH_2_–) protons within triglycerides and esterified cholesterol. In isolation, increased lipid2 may therefore suggest increased proportion of saturated fatty acids in serum. This could possibly result from oxidative stress from chemotherapy,^39^ causing selective degradation of polyunsaturated lipids, as these are more easily subject to peroxidation.^40^ An alternative hypothesis could be that unsaturated fatty acids may be consumed for membrane re-synthesis during chemotherapy-induced oxidative stress.^41^

Inflammation is known to cause changes in lipid metabolism, including increased levels of triglycerides/VLDL, decreased mean LDL particle size, triglyceride enrichment of lipoproteins and increased levels of sphingolipids and oxidized lipoproteins in serum.^42^ Sphingolipids are a class of lipids with important functions in cell structure and signalling, and have been implicated in the pathogenesis of metabolic syndrome.^43^ They originate from long-chain saturated fatty acids, and may be increased by a variety of stressors, including chemotherapy, oxidized LDL and excess substrate.^44–46^ Elevated sphingolipids is thought to contribute to chemotherapy cytotoxicity through potentiating signalling events that drive apoptosis, autophagy and cell cycle arrest,^47^ but may also contribute to insulin resistance.^48^ Chemotherapy has been shown to affect lipid profiles in breast cancer patients,^5,49,50^ causing increased LDL and reduced HDL. The observed changes in our study are consistent with inflammatory effects, and support the hypothesis that chemotherapy, in addition to producing desired cytotoxic effects, may also impair insulin sensitivity and increase atherogenesis. The residual elevation of small LDL and HDL particles which remained 12 months after therapy cessation may indicate a long-term effect on lipid profiles.^51^

Interestingly, the lipoprotein profiles of patients who did not receive chemotherapy were also affected, both 6 and 12 months after surgery. While VLDL-associated lipids and apolipoproteins were generally increased, the increase was smaller and more variable compared with chemotherapy recipients. Triglycerides were not increased, and LDL-associated lipids and apolipoproteins were decreased, with no apparent pattern of triglyceride enrichment. This suggests that patients not receiving chemotherapy also undergo significant changes in lipoprotein profiles, which differ from those seen in chemotherapy recipients. This could be caused by other treatment modalities, such as radiation or endocrine therapy, or associated changes in lifestyle and exposures over the course of treatment. It is also possible that there is a metabolic effect of surgery, proportional to the tissue damage inflicted during the procedure. After 12 months, lipids and apolipoproteins in VLDL were increased, while those associated with LDL and HDL were decreased, with the exception of triglycerides. These changes resembled those seen in chemotherapy recipients, but were not significant in univariate analyses, and no increase in triglycerides or small dense LDL particles was seen.

Patients with increased risk of weight gain had lower levels of acylcarnitines (ACs), lyso-phosphatidylcholines (LPCs), phosphatidylcholines (PCs) and sphingolipids before treatment. Few studies have examined the association between these metabolites and risk of weight gain in humans. One study found that weight gain during treatment was associated with higher baseline lactate and alanine, as well as higher body fat.^19^ We could not reproduce this, possibly because we also included women not receiving chemotherapy. Reduced levels of PCs has been associated with risk of abdominal weight gain and type 2 diabetes.^52,53^ PC supplementation has been shown to alleviate diet-induced obesity and hepatic steatosis in mice,^54^ suggesting a protective role for these metabolites. LPCs have however shown contradicting associations with obesity; LPCs have been found to be decreased in obesity in several studies,^55,56^ but were found to be increased in obese monozygotic twins compared to their non-obese twin.^57^ Twin studies allow for eliminating the influence of genetics, and increased LPCs in this study was therefore due to environmental factors. Decreased LPCs in future weight gainers may therefore be caused by genetic factors contributing to obesity.

We found that patients at risk of weight gain had lower levels of ACs at baseline, and higher levels after 6 months. ACs are produced to enable the transport of the fatty acyl groups past the mitochondrial membrane for β-oxidation.^58^ ACs are increased in obesity and type 2 diabetes, possibly due to excessive β-oxidation, which outpaces tricarboxylic acid cycle capacity.^59^ ACs have also been found to increase in mice in response to diet high in fat and sugar, concurrently with systemic mitochondrial dysfunction and weight gain.^60^ ACs may therefore be reflective of the relationship between fatty acid availability, β-oxidation activity and oxidative phosphorylation. Mice with deficient lipolysis gain more weight in response to a high-fat diet, and in vitro adipocyte lipolytic activity has been found to be negatively associated with weight gain in humans.^61,62^ Decreased lipolysis might reduce availability of substrates, possibly explaining the global reduction in lipid substances observed in patients at risk for weight gain. However, studies relating plasma lipidome to lipolytic rate and risk of weight gain are needed.

Weight gainers also had a significantly different lipoprotein profile at baseline, compared with non-weight gainers. This group had a similar lipoprotein profile to chemotherapy recipients, characterized by higher levels of VLDL-associated lipids and apolipoproteins, and lower levels of LDL 1–3- and HDL 1–3-associated lipids and apolipoproteins, with a triglyceride enrichment pattern in HDL. There is evidence that low-grade inflammation contributes to weight gain,^63^ which may explain why our patients displayed an inflammatory lipid profile at baseline, although no relation was seen after 6 months.

Our study has some limitations. The time from cessation of chemotherapy to blood sampling may vary between patients. Because our patients went through multiple types of treatment, there is a potential for confounding. Because of small group sizes, we did not perform additional subgrouping based on type of chemotherapy, radiotherapy and endocrine therapy. We did not consider more rigorous validation procedures, such as double cross-validation to be feasible for this group size. At the time of analysis, our patients were participating in a physical exercise intervention in which they were randomized to an intervention group or control group. Exercise is known to affect metabolic profile and inflammatory markers.^64^ However, as the two groups were randomized by menopausal status, and equally distributed in recipients and non-recipients of chemotherapy, we believe that the observed differences between the groups are not attributable to the intervention. The main strengths of the study are the longitudinal design, which minimizes potential effects of baseline differences between treatment groups, and standardized sampling conditions, including fasting blood samples.

In summary, our data suggest that serum metabolites and lipoprotein profiles are significantly affected during breast cancer therapy. Our findings suggest that chemotherapy recipients experience changes in lipid metabolism, leading to a transient inflammatory lipid profile consisting of high triglycerides, VLDL, increased small LDL, low HDL, triglyceride enrichment of LDL and HDL and increased sphingolipids. They also experienced increases in kynurenine and ADMA levels, which could possibly affect treatment response, risk of recurrence and cardiovascular health. These metabolic changes could potentially be connected to chemotherapy-induced oxidative stress and inflammation. While it is well established that chemotherapy reduces the risk of recurrence^65^, it should be studied whether risk of recurrence is related to metabolite profile and the metabolic response to treatment. We also observe that weight gain during breast cancer treatment may be associated with decreased acylcarnitines, lyso-phosphatidylcholines, phosphatidylcholines and sphingolipids at baseline, suggesting alterations in lipid metabolism in these patients. Our findings need to be validated in larger studies.

## Electronic supplementary material

Supplementary table 1

Supplementary table 2

Supplementary Table 3

Supplementary figure 1

Supplementary figure 2

Supplementary figure 3

Supplementary figure 4

Supplementary Figure 5

Titles and legends for supplementary figures

## **Data availability:**

The metabolomics data can be made available from the authors upon request.
