# Supplementary figures and images for: Metabolite and lipoprotein responses and prediction of weight gain during breast cancer treatment
Source: Br J Cancer. 2018 Nov 7;119(9):1144–54. doi: 10.1038/s41416-018-0211-x (PMC6220113; doi:10.1038/s41416-018-0211-x)

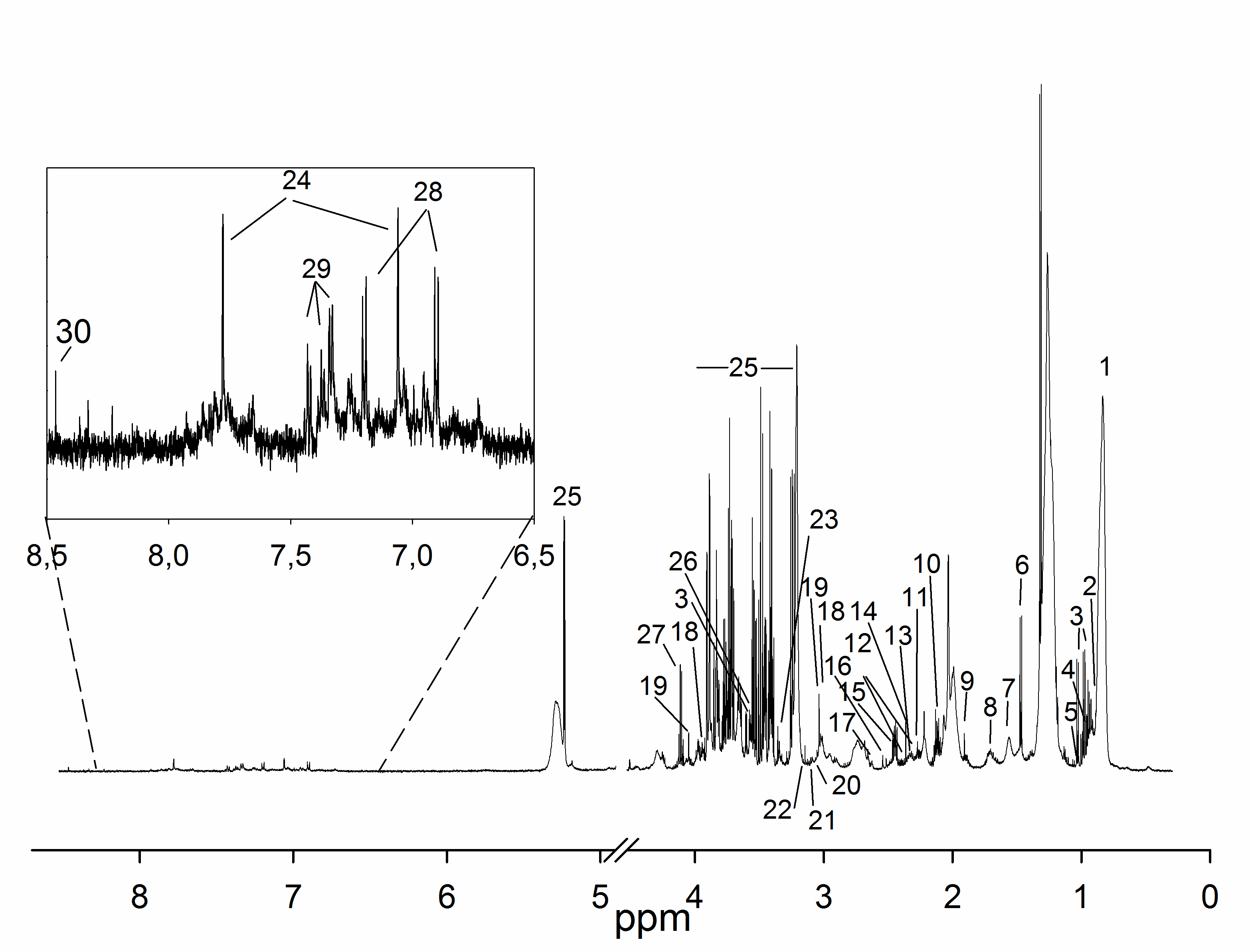

Supplement: Supplementary file 4 — Supplementary figure 1 [file 41416_2018_211_MOESM4_ESM.tif]

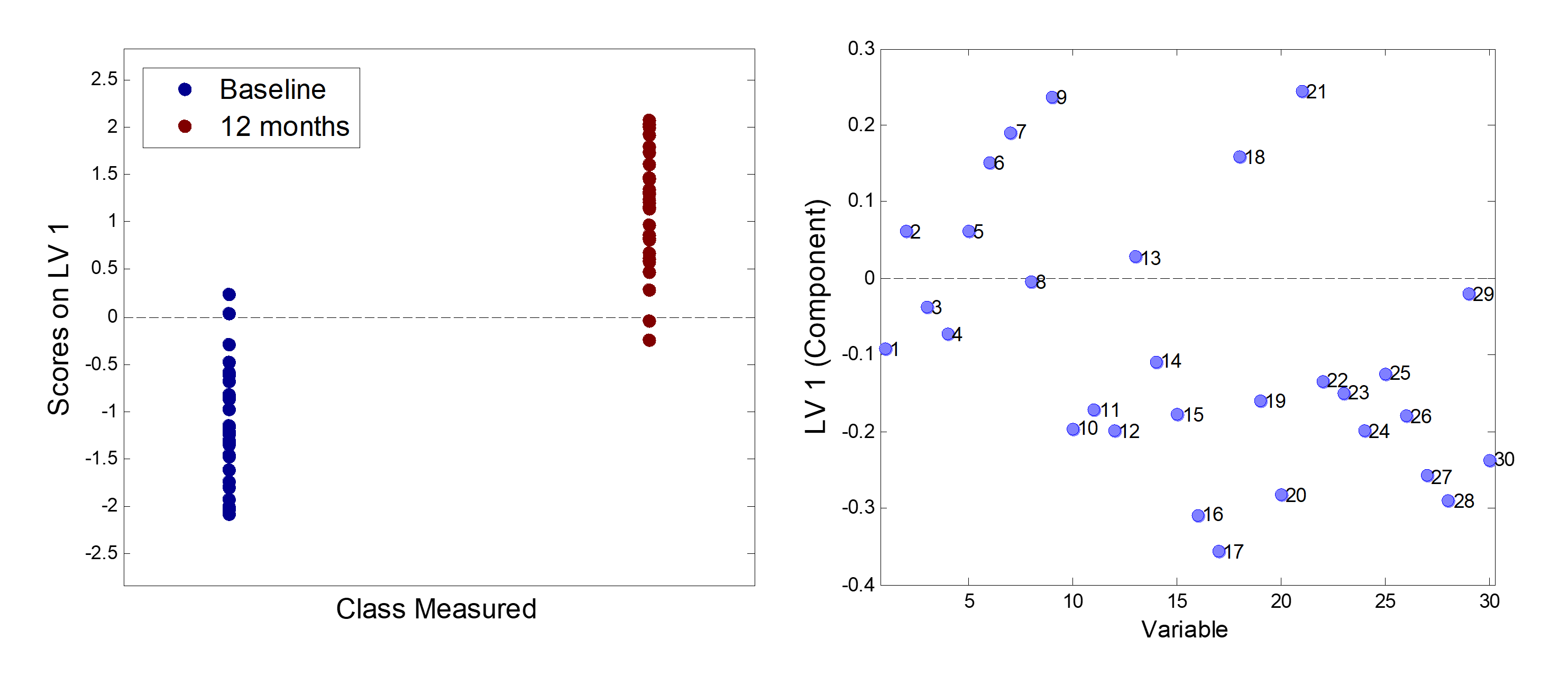

Supplement: Supplementary file 5 — Supplementary figure 2 [file 41416_2018_211_MOESM5_ESM.tif]

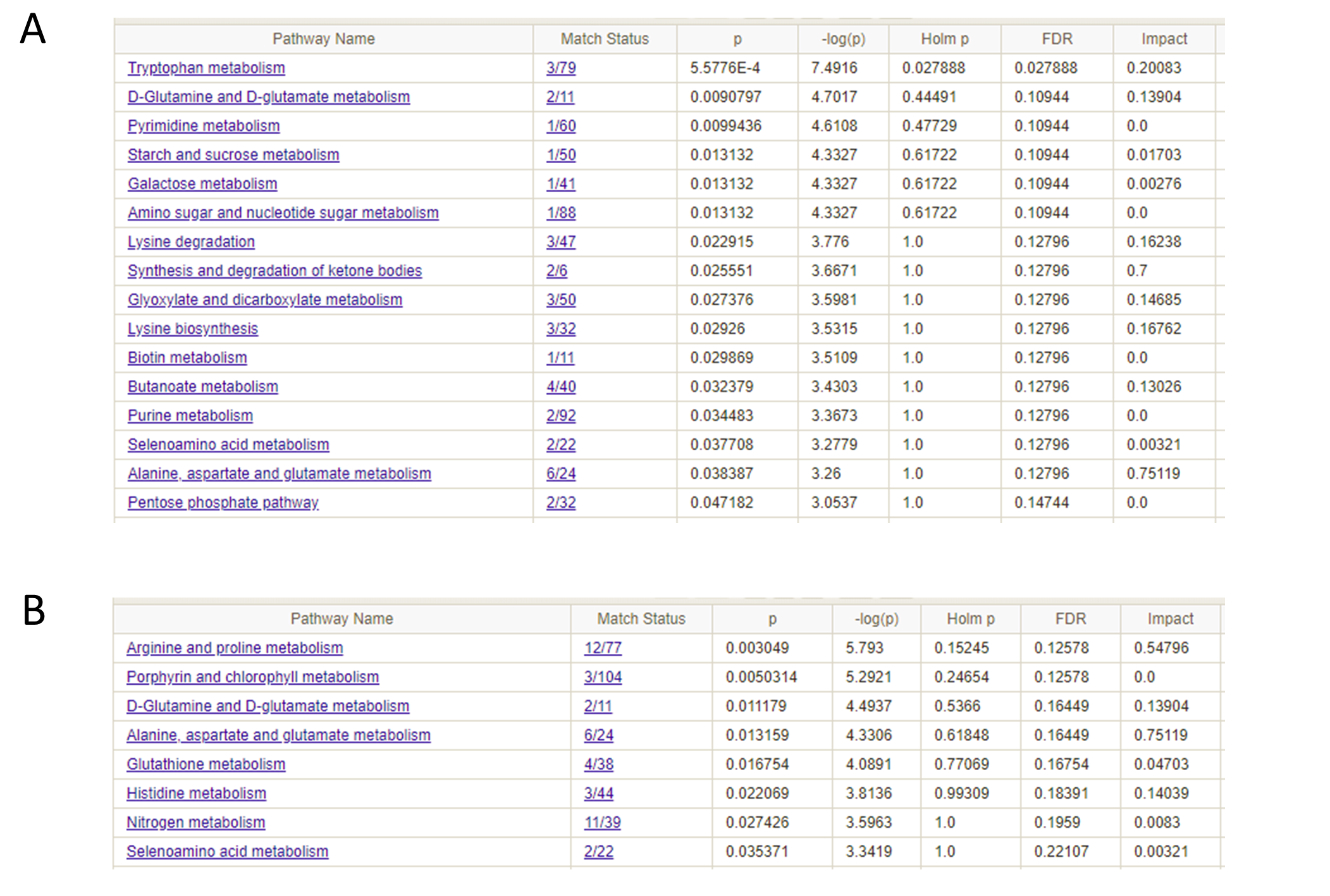

Supplement: Supplementary file 6 — Supplementary figure 3 [file 41416_2018_211_MOESM6_ESM.tif]

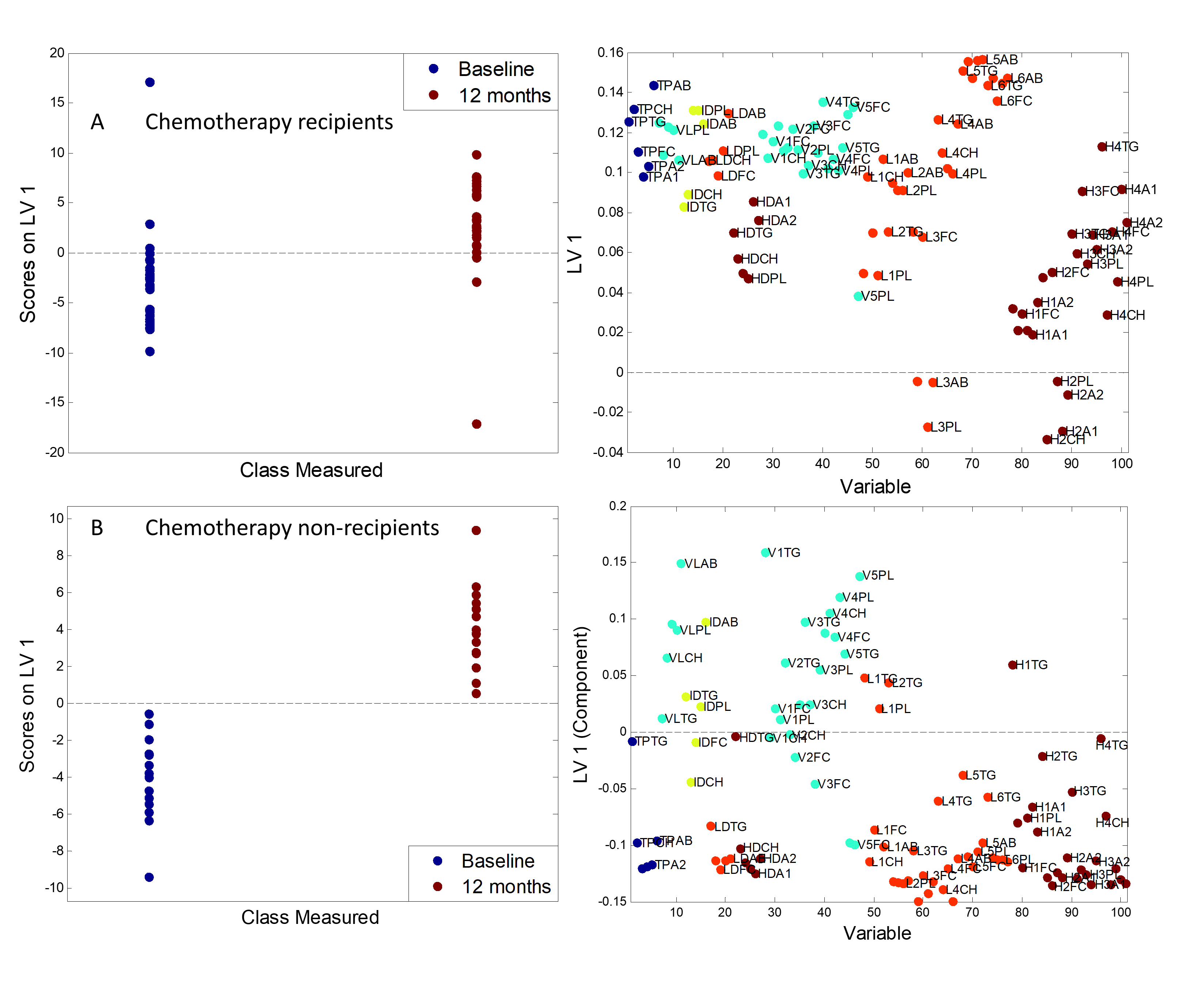

Supplement: Supplementary file 7 — Supplementary figure 4 [file 41416_2018_211_MOESM7_ESM.tif]

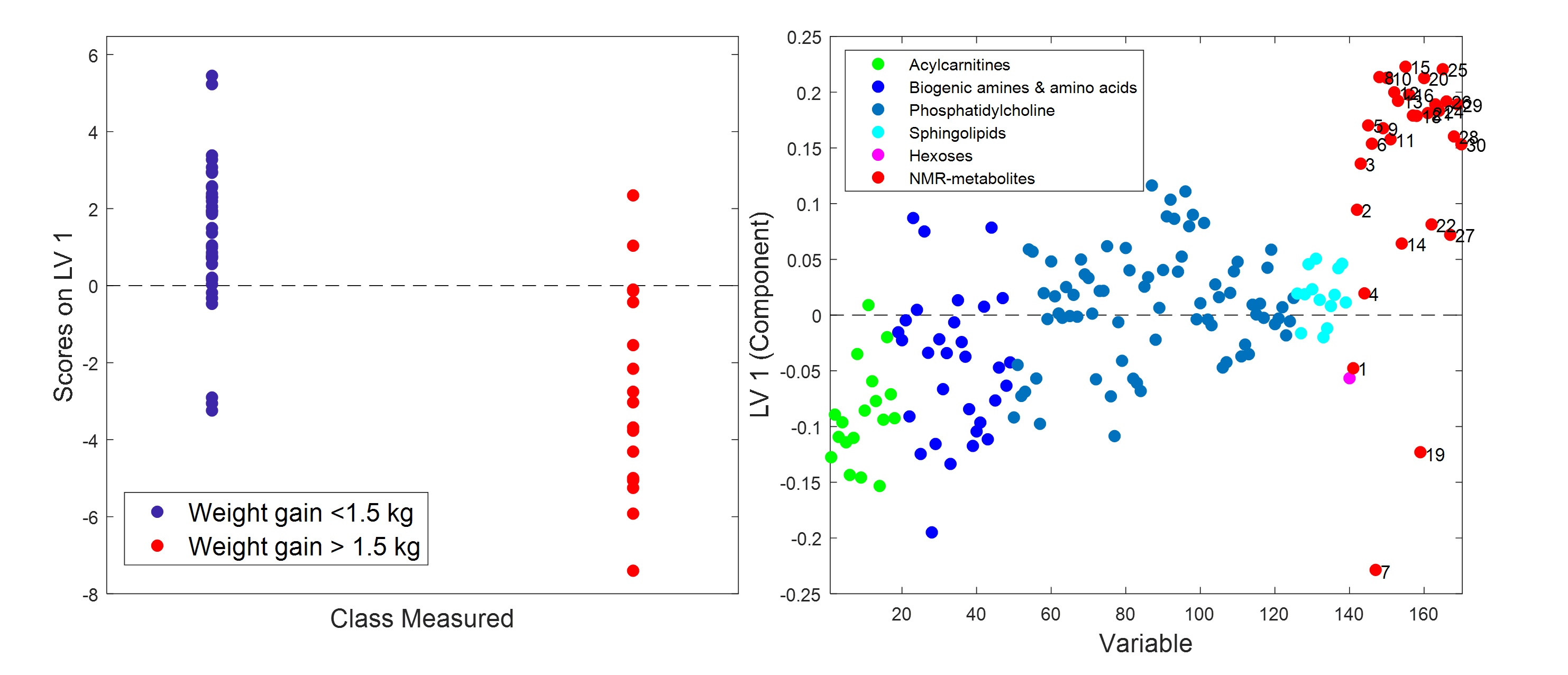

Supplement: Supplementary file 8 — Supplementary Figure 5 [file 41416_2018_211_MOESM8_ESM.tif]
