# Supplementary material for: Metabolite and lipoprotein responses and prediction of weight gain during breast cancer treatment
Source: Br J Cancer. 2018 Nov 7;119(9):1144–54. doi: 10.1038/s41416-018-0211-x (PMC6220113; doi:10.1038/s41416-018-0211-x)
Supplement: Supplementary file 9 — Titles and legends for supplementary figures [file 41416_2018_211_MOESM9_ESM.docx]

**Titles and legends for supplementary figures**

**Supplementary Figure 1:** Representative NMR-spectrum with annotated metabolite peaks. See Supplementary Table 1 for peak identities.

**Supplementary Figure 2:** Score- and loading plots for multilevel OPLS-DA between baseline and 12 months in chemotherapy recipients using metabolite profile measured by MRS. MRS-metabolites are labeled: lipid1 (1), leucine (2), valine (3), isoleucine (4), 2-methylglutarate (5), alanine (6), lipid2 (7), lysine (8), acetate (9), glutamine and glutamate (10), acetoacetate (11), 3-hydroxybutyrate (12), glutamate (13), pyruvate (14), glutamine (15), citrate (16), methionine (17), creatine (18), creatinine (19), ornithine (20), proline betaine (21), dimethyl sulfone (22), unknown (23), histidine (24), glucose (25), glycine (26), lactate (27), tyrosine (28), phenylalanine (29), formate (30).

**Supplementary Figure 3:** Results from MetaboAnalyst pathway analysis comparing metabolite profiles at baseline and 6 months for patients receiving (A) and not receiving (B) chemotherapy. Only pathways with significant p-values before multiple testing correction are shown.

**Supplementary Figure 4:** Score- and loading plot for multilevel PLS-DA of chemotherapy recipients (A) and non-recipients (B) from baseline to 12 months. See methods section for lipoprotein nomenclature. In (A), LDL5-6-associated lipids are the most highly increased, and a shift towards smaller HDL is observed.

**Supplementary Figure 5:** Score- and loading plot for prediction of weight gain using metabolite profiles after six months. Patients who gained weight had higher levels of acylcarnitines, lipid2 and isoleucine after six months.
